# The Optical Properties of Leaf Structural Elements and Their Contribution to Photosynthetic Performance and Photoprotection

**DOI:** 10.3390/plants10071455

**Published:** 2021-07-15

**Authors:** George Karabourniotis, Georgios Liakopoulos, Panagiota Bresta, Dimosthenis Nikolopoulos

**Affiliations:** 1 Laboratory of Plant Physiology and Morphology, Faculty of Crop Science, Agricultural University of Athens, Iera Odos 75, 118 55 Athens, Greece; gliak@aua.gr (G.L.); d.nikolopoulos@aua.gr (D.N.); 2 Laboratory of Electron Microscopy, Faculty of Crop Science, Agricultural University of Athens, Iera Odos 75, 118 55 Athens, Greece; brestapan@aua.gr

**Keywords:** bundle sheath extensions, epidermis, leaf anatomy, light harvesting, mesophyll, optical properties, photoprotection, photosynthesis, trichomes, UV radiation

## Abstract

Leaves have evolved to effectively harvest light, and, in parallel, to balance photosynthetic CO_2_ assimilation with water losses. At times, leaves must operate under light limiting conditions while at other instances (temporally distant or even within seconds), the same leaves must modulate light capture to avoid photoinhibition and achieve a uniform internal light gradient. The light-harvesting capacity and the photosynthetic performance of a given leaf are both determined by the organization and the properties of its structural elements, with some of these having evolved as adaptations to stressful environments. In this respect, the present review focuses on the optical roles of particular leaf structural elements (the light capture module) while integrating their involvement in other important functional modules. Superficial leaf tissues (epidermis including cuticle) and structures (epidermal appendages such as trichomes) play a crucial role against light interception. The epidermis, together with the cuticle, behaves as a reflector, as a selective UV filter and, in some cases, each epidermal cell acts as a lens focusing light to the interior. Non glandular trichomes reflect a considerable part of the solar radiation and absorb mainly in the UV spectral band. Mesophyll photosynthetic tissues and biominerals are involved in the efficient propagation of light within the mesophyll. Bundle sheath extensions and sclereids transfer light to internal layers of the mesophyll, particularly important in thick and compact leaves or in leaves with a flutter habit. All of the aforementioned structural elements have been typically optimized during evolution for multiple functions, thus offering adaptive advantages in challenging environments. Hence, each particular leaf design incorporates suitable optical traits advantageously and cost-effectively with the other fundamental functions of the leaf.

## 1. Introduction

In order for plants to achieve a positive balance of energy and carbon, four key leaf modules, mostly located in plant leaves, i.e., the light capture module, the water–nutrient flow module, the gas exchange module, and the defense (against biotic stresses)–protection (against abiotic stresses) module have to collaborate [1]. Each module consists of a number of structural elements (tissues and/or organs) either for the acquisition of resources (light energy, water, nutrients, and CO_2_) or for protection and defense in a given environment.

Leaf functions must be harmonized with all environmental variables, including the light regime, in order for photosynthesis to proceed under favorable circumstances. At times, leaves must operate under light limiting conditions, meaning that they must maximize light capture, while at other instances the same leaves may function under very strong light and must modulate light capture to avoid photo-inhibition [2]. These contrasting conditions may occur hours or even seconds apart. Finally, for the optimization of photosynthetic assimilation, the light inside the mesophyll should be distributed both deeply and uniformly [3,4].

**Figure 1:**
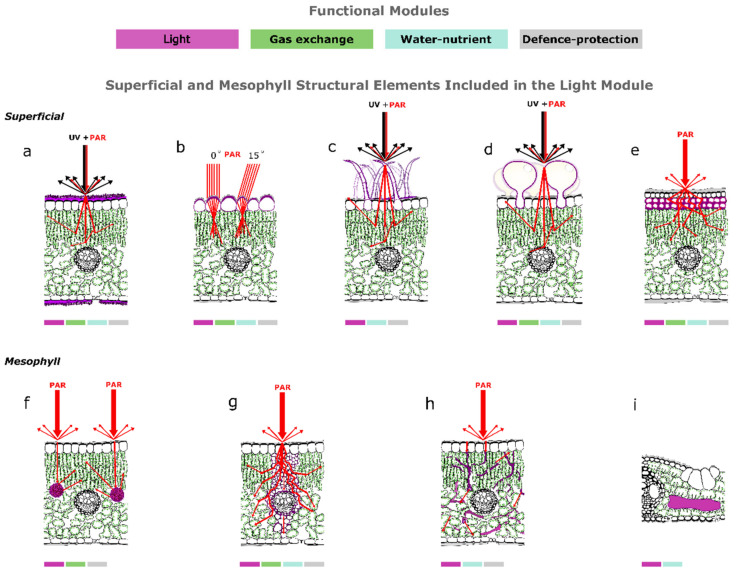
Conceptual representation of the optical properties of the nine (**a–i**) structural elements of leaves (typical bifacial leaves with various structural idiosyncrasies (**a**–**h**) and representative bamboo leaf (**i**)) included in the light module: (**a**) cuticle light reflectance, (**b**) epidermal cell light focusing, (**c**) non-glandular hair light reflectance and scattering, (**d**) gland light reflectance and scattering, (**e**) multiseriate sclerified hypodermis light scattering, (**f**) crystal or cystolith light scattering, (**g**) bundle sheath extension light scattering and path lengthening, (**h**) sclereid light transfer, and (**i**) bamboo’s fusoid cell. Five elements are superficial (**a–e**) and four elements are located in the mesophyll (**f–i**). The structural elements presented are highlighted in magenta in each drawing. In each structural element, arrows (red for photosynthetically active radiation (PAR) and black for ultraviolet radiation (UV)) show surface light reflectance, surface and mesophyll light scattering, and hypothesized light path lengthening. Light absorption phenomena (e.g., UV radiation absorption from epidermal cells in (**a**)) are omitted for clarity. The optical properties for bamboo fusoid cells (**i**) were not conceptualized due to the unavailability of data from the literature. Colour coding represents the four functional modules of leaves (light, gas exchange, water–nutrient, and defense–protection modules) and coloured rectangles under each drawing show all the functional modules in which each element participates.

The strong variability of the structural elements comprising the light capture module (Figure 1) has led to a large diversity in leaf designs during plant evolution, despite functional elements, such as photosynthetic metabolism, having remained remarkably conserved throughout phylogeny [5,6]. This tremendous diversity of leaf anatomical and physical properties manifests both the necessity for adaptability to different environments and the strong influence of these properties on photosynthesis itself. The evolutionary direction of the different leaf designs is not the optimization of a single structural or functional element, but the refinement of either a combination or a collaboration of the different elements in order to successfully adapt to the particular environment [1]. Regarding the light capture module, the properties and the organization of the structural elements of the lamina determine the light-harvesting capacity and the photosynthetic potential of the leaf [6]. This organization creates a leaf design adapted to a particular growth environment that combines the suitable optical traits with the functions of the other three modules. For example, the high ultraviolet (UV) and photosynthetically active radiation (PAR) intensities in xeric environments require suitable optical protective elements that have to be compatible with effective CO_2_ acquisition and minimal water losses. In contrast, the very low energy supply in the forest understory requires suitable optical elements for effective maximal light harvesting.

In this review we examine the following: (1) the optical role of specific leaf structural elements within the frame of the light capture module and (2) the potential involvement of these elements in the function of the other three modules, as well as their probable combination with other leaf structural traits.

## 2. Superficial Structural Elements and Light Interception

The leaf surface has a key role in protection against multiple stress factors such as water loss, insect or pathogen attack, excess PAR and UV radiation. and overheating. Superficial leaf tissues (epidermis including cuticle) and structures (epidermal appendages such as trichomes and glands) play a crucial role against light interception [7] (Figure 1a–e). As they comprise the outermost boundary receiving the incident light, their optical properties determine the reflectance, absorbance, and transmittance of the leaf, and thus the light quantity (intensity) and quality (spectral distribution) reaching photosynthetic tissues. Hence, the leaf surface shows a great heterogeneity in terms of optical properties, depending not only on the particular species, but also on environmental conditions. For example, the optical characteristics of the leaf surface and thus the leaf reflectance spectra are species-specific and are related to the evolutionary dynamics of the leaf chemistry and structure, and thus to the phylogenetic history of each plant [8]. Furthermore, acclimatization responses may also account for changes in the optical properties of leaf surfaces (see below 2.1 to 2.8).

### 2.1. The Epidermis Is a Selective Optical Filter

The epidermis is usually one compact layer of chloroplast-free cells without intercellular spaces. The outer walls of epidermal cells are overlaid by the structurally complex layer of the cuticle, which consists mainly of polymerized lipid materials such as cutin or cutan and intra- and epi-cuticular waxes [9,10,11]. The cuticle and epidermis are virtually transparent to PAR, but absorb in the UV-B spectral band (280–315 nm) [12,13,14,15,16]. Despite UV-B being a minor and highly variable fraction of the incident solar radiation, the photons of this spectral region are the most energetic of those reaching the Earth’s surface and cause irreversible photochemical reactions such as bond cleavage, oxidation, dimerization, and free radical generation causing damage to biological molecules, such as DNA, RNA, and proteins [17,18,19]. Plants are unavoidably exposed to solar UV-radiation because they are usually exposed to direct sun light, an inevitable condition for the growth and survival of photoautotrophs which cover virtually all of the Earth’s terrestrial surface. The epidermis, together with the cuticle, behaves as a selective optical filter that excludes damaging UV wavelengths from reaching sensitive mesophyll tissues. Phenolic compounds covalently bound to cuticular components and epicuticular waxes are mainly responsible for the UV screening ability of the cuticle [20,21,22,23,24,25,26]. The important UV screening capacity of epidermal cells is mainly provided by the accumulation of soluble phenolic compounds such as glycosylated flavonoids, hydroxycinnamic acids, anthocyanins, and, in some cases, tannins in the vacuoles of epidermal cells [27,28,29,30]. Thus, the leaf epidermis and the cuticle build up a strong barrier to incident UV-B, so that its photons are attenuated before entering the mesophyll tissues [20] (Figure 1a).

It should be pointed out that the UV-screening function of epidermal phenolic compounds is only one of the multifaceted roles of these metabolites. Photo-protection itself is also provided by the radical scavenging ability of phenolic compounds [30], while it should be noted that their strategic localization at the plant surface exerts additional roles related to biotic stressors [31].

### 2.2. The Epidermis Is a Reflector

The epicuticular waxes and the cuticle covering the surface of all aerial plant parts reflect both UV and PAR, but not necessarily to the same extent, with UV (and blue) reflectance being greater in some cases [32] due to Rayleigh scattering, owing to the molecular composition and fine-structure modifications of elements of superficial structures such as the layer of epicuticular waxes [3,33]. The shape, diameter, and distribution of the epicuticular wax crystals covering the leaf surface strongly affect the magnitude and spectral characteristics of reflectance [34]. “Glabrous” (smooth) cuticles typically show limited reflectance (less than 10%), despite the fact that their (specular) reflection presupposes the opposite, while “glaucous” (waxy) ones show considerably higher reflectance (30% or more), both in the UV and photosynthetically active spectral bands, virtually as scattering [26,35]. The magnitude of reflectance is also affected by the angle of incidence of (collimated) light. It is expected that when the angle is low (i.e., more oblique; taken that perpendicular illumination angle is at 90° to the leaf plane), reflectance is increased [36]. In leaves of monocotyledons that show axial symmetry, the geometrical arrangement between the leaf axis and the direction of light incidence may also affect reflectance, as well as other optical phenomena related to light interception [37].

While UV reflectance appears to occur from the cuticle–air interface, PAR reflectance appears largely from the mesophyll as a result of diffused internal reflectance [38,39]. As such, it shows a characteristic spectral composition owing to the selective absorption of red and blue wavebands from photosynthetic pigments [33]. The reflecting capacity of the epicuticular waxes can protect epidermal cells themselves, whereas the above-mentioned filtering capacity of the epidermis protects mainly the underlying photosynthetic tissues against UV radiation damage [19] (Figure 1a).

### 2.3. Phytoliths Protect the Underlying Tissues from Photo-Inhibition

Silicon (Si) is a key beneficial structural element in grasses as it enhances the leaf strength and mitigates various stress effects. Si is deposited in the form of phytoliths in the epidermis of the leaves, including epidermal long cells, bulliform cells, guard cells, and prickle hairs. Although there is no direct evidence, there are some indications that these structural elements can reflect the incident UV radiation, thereby protecting the underlying tissues from photo-inhibitory damage [40,41,42,43]. The question of whether phytoliths modulate the light microenvironment of the mesophyll has received a negative answer so far [44].

### 2.4. The Epidermal Cells of Understory Plants Focus Light

The shape of epidermal cells affects the geometry of the light entering a leaf, depending on the curvature of the outer epiclinal cell wall, which resembles the entry surface of a convex lens. In some plant species, the adaxial epidermal cells are papillose and the outer walls of these cells are unusually lens-shaped, protruding from the leaf surface [45] (Figure 1b). These characteristics are frequently found in understory tropical species such as *Anthurium* and *Begonia*. Understory plants grow on the forest floor beneath a dense canopy where the light regime is typically diffuse and photon flux density (PFD) values are very low, even to attain gross photosynthetic rates higher than the light compensation point. Direct light can penetrate to the understory only in the form of sunflecks. Aside from these intense rays of direct light, the photosynthetic performance of understory plants relies mainly on low PFD diffuse light. These epidermal cells behave as convex lenses concentrating collimated light (such as direct sunlight) in the mesophyll area (Figure 1b) (for recent reviews, see [46,47]). Light focusing may serve to increase the photosynthetic rate of the mesophyll cells [48,49], primarily when sunflecks penetrate to the ground level of the forest [50]. The roundedness of epidermal cells affects the degree of light focus [4,51]. There is evidence, however, that contrary to the initial hypotheses, lens-shaped epidermal cells do not contribute significantly to harvesting diffuse light [4,50]. There is an open question, therefore, concerning the contribution of these structural elements to the photosynthetic performance of leaves.

### 2.5. Epidermal Windows Enable Underground Photosynthesis in Succulents

Succulent species of the genus *Lithops* (living stones), native in South Africa, display some of the most peculiar structural and functional adaptations in the plant kingdom to tolerate adverse conditions (drought, high temperatures, and high light intensity) in their growth environment. The major part of the biomass of these plants, including much of their photosynthetic tissue, is located underground [52]. The exposed epidermis and the underlying water-storing, non-chlorophyllous parenchyma tissue of the leaf tips of these plants are transparent or translucent, allowing light penetration deep into the below ground part of the thick leaves where the photosynthetic cells are located. These structural elements are often called “epidermal windows”, and may be the only plant part visible at the ground surface [53,54]. Thus, this subterranean photosynthesis functions in an environment where light energy is typically unavailable, but it is a cooler and more stable environment compared with the atmospheric one. Moreover, the suitable design and pigmentation of the leaf tips of *Lithops* protects the plant from herbivory by small mammals through camouflage. Light enrichment through the epidermal windows is expected to benefit carbon assimilation. However, covering these windows did not seem to reduce net assimilation rates in three succulent species [55]. The presence of large epidermal windows can cause photoinhibition, because of the increased internal leaf temperatures due to the greater penetration of the infrared spectral region (see also [56]). This may explain the controversial results showing a lack of difference between the covered and uncovered windows [55], as the reduction of light penetration might be compensated by the development of lower internal temperatures that are more favourable for photosynthesis [57]. At the interspecific level, the size of the epidermal windows correlates inversely with the solar irradiance of the growth environment. Species with large windows thrive in cloudy, high-rainfall regions, whereas species thriving in high solar irradiance regions have small windows, minimizing the probability of photo-inhibition [52]. Moreover, *Lithops* plants have sufficient biochemical flexibility to respond to variable light conditions within the same leaf (extreme high light intensity in the above ground region and moderate or low intensity in the below ground) [58,59].

### 2.6. Non Glandular Trichomes Function as Reflectors and UV Screens

Trichomes (or hairs) are unicellular or multi-cellular superficial appendages of an epidermal origin, classified either as glandular or non-glandular [60]. Glandular trichomes secrete or store large quantities of materials of a diverged origin, such as excess sea salt or lipophilic mixtures of secondary metabolites, such as terpenoids and phenolics [60,61]. Non-glandular trichomes do not possess a secretory function, but usually create dense layers (indumenta) on the surfaces of plant organs. These layers display discrete optical properties. They act as shields against harmful wavelengths, offering protection to the underlying leaf tissues against UV-B radiation (Figure 1c) (for a recent review see [62]). Experiments with fibre-optic microprobes confirmed that the trichome layers of olive and holm oak leaves attenuate almost all incident UV-B (at 310 nm) and UV-A (at 360 nm) radiation and a considerable portion of blue light (at 430 nm) [63,64]. Moreover, the density of the trichomes is negatively correlated with sensitivity to UV-B radiation, further suggesting the UV-protective role of these structural elements [65,66]. The UV absorbing capacity of trichomes is attributed to the diffused deposition of phenolic compounds (especially flavonoids) in their cell walls [62,67,68,69,70,71,72]. Dense indumenta also act as reflectors, reducing the radiant energy absorbed by the leaf lamina [73,74]. The above-mentioned light filtering and reflecting properties of the trichome layers may also afford protection against high light intensities causing photo-inhibition, especially in young leaves [63,64,75,76,77,78] (Figure 1c). Consequently, trichome layers may reduce light harvesting for photosynthesis under low irradiance or low angles of incidence. They may also affect the geometry of collimated direct light by transforming it to diffuse light. Trichome may, therefore, affect other optical properties of leaf surfaces such as epidermal focusing ([50]; see Section 2.4, above) or the propagation of light into the mesophyll, again affecting the profile of the internal light microenvironment and the degree of light saturated photosynthesis of the internal cell layers [79,80,81].

### 2.7. Superficial Salt Crystals Excreted by Glands Function as UV Screens and Reflectors

Recretohalophytes are halophytes able to secrete salt out of the leaf interior directly onto the leaf surface, due to the occurrence of superficial salt glands [82]. The excretory function of these glands may indirectly affect the optical properties of the leaf surface by reflecting part of the incident radiation, reducing photodamage and overheating during stressful periods [83,84] (Figure 1d).

### 2.8. Hypodermal Sclerenchymatic Tissues Protect Mesophyll from UV and Par Damage

**Figure 2:**
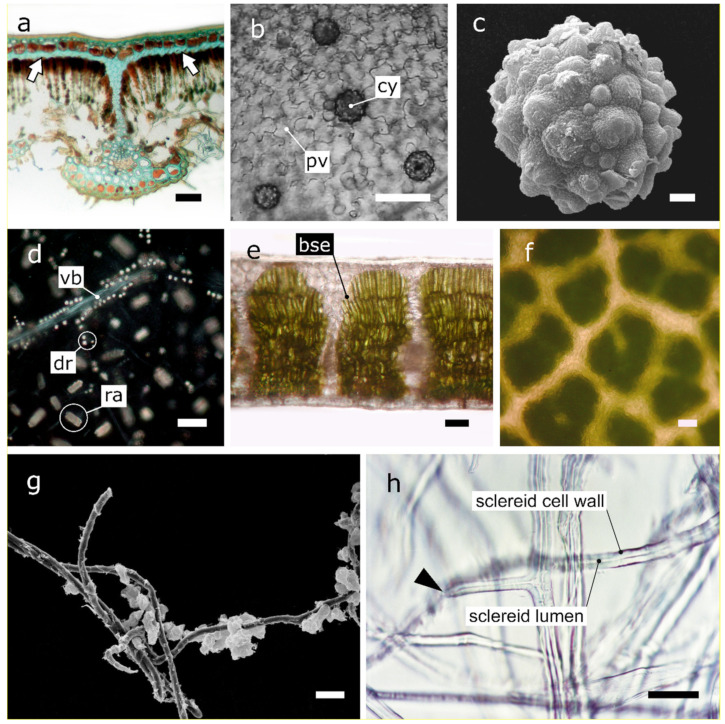
In planta or isolated mesophyll structural elements. (**a**) Light microscope cross section view of sclerified pseudohypodermis of a *Banksia marginata* leaf. Photograph kindly provided by Prof. G.J. Jordan; see also [86]. (**b**) Light microscope paradermal view of cystoliths (cy) of a *Parietaria judaica* leaf bleached with sodium hypochlorite solution. Pavement cells (pv) of the adaxial epidermis are also visible. Photograph reproduced from [95]. (**c**) Electron scanning microscope view of an isolated cystolith from a *Parietaria judaica* leaf. Photograph reproduced from [95]. (**d**) Polarized light microscope paradermal view of a *Vitis vinifera* developing leaf bleached with a sodium hypochlorite solution. Crystals of calcium oxalate (raphides (ra) and druses (dr)) are visible on a dark background. Note the numerous druses arranged along the vascular bundle (vb). Photograph by A. Giannopoulos; unpublished. (**e**) Light microscope cross section view of a *Quercus coccifera* leaf. Bundle sheath extensions (bse) are seen as translucent areas between areoles. Photograph by V. Liakoura; unpublished. (**f**) Light microscope paradermal view of a *Quercus coccifera* leaf showing the network of bundle sheath extensions. Photograph by V. Liakoura; unpublished. (**g**) Electron scanning microscope view of enzymatically isolated sclereids from an *Olea europaea* leaf. Some spongy parenchyma cells are still attached on the sclereid. Photograph by C. Fasseas and G. Karabourniotis; unpublished. (**h**) Light microscope view of enzymatically isolated sclereids from an *Olea europaea* leaf. Note the anatomical resemblance of sclereids (cell wall and lumen) to optical fibres (cladding and core, respectively). An intact end point is visible (arrowhead). Photograph by G. Karabourniotis; unpublished. Scale bars (**a**,**b**,**d**–**h**: 50 μm; **c**: 5 μm).

Species of the major Southern Hemisphere family, Proteaceae, are characterized by sclerophyllous leaves with very thick cuticles and multiseriate sclerified pseudohypodermis beneath the epidermis and hypodermis (Figure 2a). Based on the fact that many of these structures are associated with the leaf surface exposed to direct light, Jordan et al. (1998) [85] proposed that they protect the mesophyll from excess solar radiation, including the photosynthetically active, UV, and possibly the infrared spectral band as well. These structural elements increase the path through which photons must travel and thus increase the attenuation of UV and PAR before reaching the mesophyll [85,86,87] (Figure 1e).

## 3. Mesophyll Structural Elements Allow Efficient Light Propagation and Internal Light Homogenization

**Figure 3:**
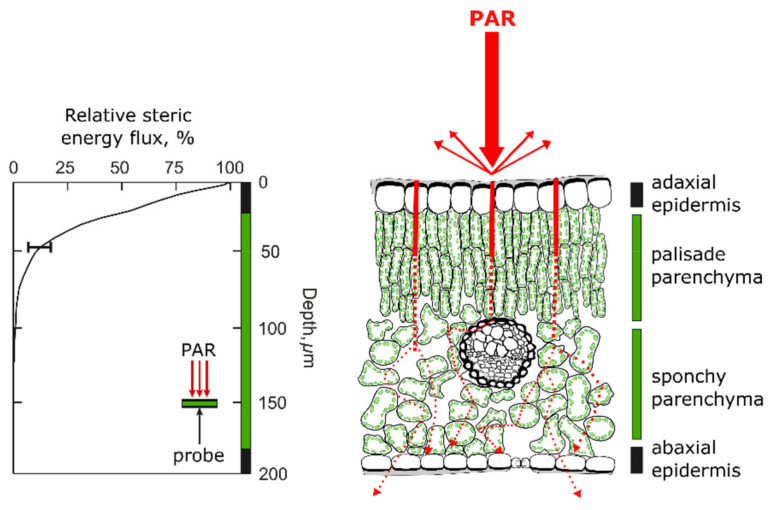
Gradient profile of blue light as relative steric energy flux, % of incident photosynthetically active radiation (PAR), inside a bifacial leaf of *Vitis vinifera* and measured using a fibre optic microprobe. The leaf was irradiated with collimated light with the adaxial surface facing the light. The probe was inserted from the abaxial leaf side and advanced directly through the leaf until reached the adaxial side, while recording the amount of light (curve in the left part of the figure). See [103] (Figure 3) for more details. (Right) Representation of a typical bifacial leaf. Red arrows show a conceptual representation of surface light reflectance, surface and mesophyll light scattering, and hypothesized light path lengthening. Red lines appear thinner along the depth representing light depletion. The vertical thick lines (left and right) give the thickness (depth) of the leaf (black epidermises; green: mesophyll tissues). Gradient light profile graph (left) is reproduced from [103].

Leaf tissues, both photosynthetic and non-photosynthetic, as well as other structural components, such as phytominerals and idioblasts, participate in the modulation of the internal light environment. Light propagation should be such as to allow for deep light penetration and a uniform light environment, i.e., form a smooth internal light gradient along the mesophyll depth. This is particularly important under conditions of strong collimated light, because such light conditions may favour steep light gradients inside the mesophyll. On the other hand, a strong incident light is a prerequisite for an optimal photosynthetic rate which, in turn, requires a smooth light gradient inside the mesophyll in order to allow all of the photosynthetic cells to photosynthesize at a quite high rate (Figure 3). The light capture module is equipped with a large array of structural elements to achieve internal light homogenization (see below; Figure 1f–i).

### 3.1. Mesophyll Cells Affect the Light Interception Efficiency of the Leaves

The optical properties of the photosynthetic cells affect the light interception efficiency of the leaves. The most prominent example is the structural design of bifacial leaves. In these leaves, the mesophyll is usually differentiated into two distinct regions, the upper palisade and the lower spongy parenchyma layer. The palisade cells are elongated, with their axis mostly parallel to the direction of the incident collimated light. This arrangement allows these cells to operate as optical fibres, facilitating light channelling deeper into the leaf [79,80,88,89]. Bifacial leaves are thought to achieve a smoother light gradient along the mesophyll depth due to the light channeling capacity of palisade cells [79,88] (Figure 3). The spongy cells are irregularly shaped, forming large intercellular air spaces that result in a greater effective light path lengthening and, subsequently, increased light absorption through multiple light scattering as the photons encounter numerous air–cell wall interfaces [90,91,92,93]. This anatomy also allows for the effective diffusion of CO_2_ from the stomata, usually located at the abaxial epidermis in bifacial leaves, to the upper palisade layer where the larger proportion of photosynthesis takes place [94].

### 3.2. Calcium-Carbon Inclusions Improve the Light Microenvironment within Leaves

As representatives of the biomineralization process, calcium oxalate (CaOx) crystals and calcium carbonate (CaCO_3_-lime) cystoliths are common cellular solid calcium-carbon inclusions in plants (CaCIs) [96,97,98]. CaCO_3_ deposition occurs mainly in four members of the order Urticales, i.e., Cannabaceae, Moraceae, Ulmaceae, and Urticaceae, as an encrustation on cell walls or in an unusual deposit called cystolith located in enlarged idioblasts, the lithocysts [96] (Figure 2b,c). CaOx crystals on the other hand are distributed among all taxonomic levels of photosynthetic organisms, located in idioblastic cells called crystal cells (Figure 2d). It has been proposed that CaCIs can improve the light microenvironment within leaves [99]. Both CaOx crystals and CaCO_3_ cystoliths are directly involved in light scattering, reducing the steep light gradient within mesophyll and thus enabling a more efficient use of the incident PAR [43,100,101,102] (Figure 1f). CaOx crystals contained in vascular bundle sheaths and other tissues such as sclerenchyma, collenchyma, or parenchyma, could also scatter light comprising a key component in the homogenization of the light gradient profile along the depth within the mesophyll [103] (see also Section 3.3 and Section 3.4, below). Microscopic observations confirmed that the spatial distribution of the CaCIs is compatible with their proposed optical function [94]. Moreover, CaOx crystals within the epidermis of *Lithops aucampiae* leaves may scatter light within the below-ground region of the leaves, thus enriching the lower tissues with photons [59]. It was also proposed that in some species thriving in extreme environments, crystal sand may provide protection against photo-inhibition by filtering and dispersing the solar irradiance and moderating the internal leaf temperature [104].

### 3.3. Bundle Sheath Extensions Function as Transparent Windows

In many plant species, the bundle sheaths of the leaves extend to both lamina surfaces, below the epidermises (Figure 2e). These extensions (bundle sheath extensions (BSEs)) consist of parenchyma, collenchyma, or sclerenchyma cells without chloroplasts [105,106]. BSEs that extend fully on both surfaces create vertical partitions that isolate the intermediate photosynthetic cells (Figure 2e,f). Thus, the mesophyll in these leaves is divided in, small or larger, depending on vein density, photosynthetic compartments termed “areoles”, or “BSEs compartments” [91]. Leaves bearing BSEs and areoles are characterized as “heterobaric”, whereas those lacking these structural elements are called “homobaric” [91,105,106,107]. BSEs, which are relatively free of intercellular spaces and photosynthetic pigments, create transparent areas in the leaf blade (up to 50% of its surface in some species), easily seen in intact leaves as a network of bright lines on a dark green background when viewed against a bright light source or under the optical microscope [91,97,105,106] (Figure 2f). Bundle sheath extensions behave as “transparent windows” transferring light to internal layers of the mesophyll, especially important in thick and compact leaves in which otherwise deep photosynthetic cell layers would be under very poor illumination conditions ([103]; see also [4]) (Figure 1g). Light enrichment of these internal layers of photosynthetic tissue increases the photosynthetic performance of each areole; i.e., photosynthetic rate per unit volume of photosynthetic tissue. This efficient design compensates for the evident loss of photosynthetic area, especially apparent in leaves with well-developed BSE networks. According to Nikolopoulos et al. [108], photosynthetic capacity per unit of photosynthetically active leaf area is correlated with the extent of the BSE network at an interspecific level. This design also gives adaptive advantages, offering a significant water economy because thicker leaves have a lower surface to volume ratio [108,109,110]. Probably for this reason, heterobaric leaf species (mainly trees) tend to be distributed in high irradiance, occasionally xerothermic environments, such as the Mediterranean forest gaps; a similar distribution characterizes species of the Proteaceae family with sclerophyllous leaves referred above [86]. In contrast, homobaric leaf species (mainly herbs) are found in shady and moist environments such as the forest understory or their biological cycle is confined within the favourable season [91,111,112,113]. The heterobaric character is also stronger at an intraspecific level as a result of acclimatization to xerothermic conditions and high light intensities. This is evident between sun (outer canopy layer) and shade grown leaves (deep canopy layers) in representative evergreen sclerophylls [108] (see Section 5, below).

Heterobaric leaves are also common in complex canopy species (trees and shrubs) of which some species bear flexible long petioles such as in grapevine (*Vitis vinifera*) and common aspen (*Populus tremula*). The leaves of many grass species are also strongly heterobaric. All of these species must benefit from this leaf design, despite the fact that leaves are not always very thick. So, there it seems possible that, in such cases, another property of heterobaric construction is advantageous. As most of these leaves move continuously, oscillate, or tremble due to the wind, the angle of incidence of solar radiation changes accordingly. The BSE network in this case may contribute to the homogenization of light harvesting both spatially (smoothing the light gradient profile along the depth of the mesophyll) and temporally (smoothing the intense variations due to oscillations of the lead lamina), thus enhancing uninterrupted photosynthesis.

The above possibility requires studying leaves under simulated oscillations and assessing the possible contribution of the heterobaric construction to light harvesting and photosynthetic performance. It has been reported that poplar leaves are efficient at utilizing light under a fluctuating and highly variable light environment, similar to that occurring naturally due to leaf fluttering or the occurrence of sunflecks [114]. Leaf flutter allows for deep light penetration and the optimization of carbon gain at the whole canopy level [115]. Top canopy leaves that flutter also show more uniform light capture compared with artificially fixed leaves at various angles in relation to the direction of light. The latter do not capture enough light quantities at lower angles of incident and unfavourable azimuthal angles, even when exposed to full sunlight [116].

### 3.4. Sclereids Function as Optical Fibres

In some xerophytes with thick and compact leaves, the improvement of the light microenvironment within deep layers of photosynthetic cells has been undertaken by diffuse sclereids dispersed in the mesophyll. Sclereids are idioblastic cells in the form of sclerenchymatous fibres with thick, highly lignified cell walls [117] (Figure 2g,h). The anatomy and orientation of the leaf sclereids of the Oleaceae evergreen sclerophyll species *Olea europaea* and *Phillyrea latifolia* are suitable to offer a light-guiding function. Light conducted through a sclereid eventually exits the structure from the exit plane at the far end, which may be located deep within mesophyll tissues (Figure 1h). Sclereids show impressive similarities to commercial optical fibres, and the propagation of light is accomplished by the difference in the refractive indices between the thick cell wall of the sclereids and the air filling the intercellular spaces [118,119] (Figure 2h). The intensity of the light transmitted through the sclereids was found to be up to 30-fold higher (reaching up to 80% of the incident radiation at the leaf surface) compared to that transmitted through the neighbouring mesophyll cells and, compared with the latter, it is enriched with photons of the red and blue wavebands [119]. Hence, high intensity, photosynthetically efficient light guided through these structures reaches light deficient internal chlorenchyma cells, improving the photosynthetic performance (Figure 1h).

### 3.5. Fusoid Cells Improve the Light Microenvironment within Mesophyll of Bamboos

Fusoid cells are large, cigar-shaped, thin-walled, transparent cells in the mesophyll of bamboo (Bambusoideae). These cells extend more or less perpendicularly from each side of a bundle sheath into the middle of the mesophyll, with their long axis parallel to the epidermis (Figure 1i). Fusoid cells are surrounded by monolayers of chlorenchyma cells. In some cases, these cells collapse, creating large intercellular spaces within mesophyll [120]. In three bamboo species, it was observed that sun leaves are smaller and thicker and lack fusoid cells, whereas the shade leaves is consistently larger and thinner with fusoid cells. Based on the strategic placement of these cells within the mesophyll, their absence from sun leaves and the optical properties of the leaves infused by mineral oil, ref. [120] it is proposed that fusoid cells play an optical role by improving the light microenvironment within the mesophyll of shade leaves (see also [46]).

## 4. The Functional Integration with the Other Modules

As noted in the introductory section, the evolution of different leaf designs is driven by the maximization of economic efficiency, achieved by a profitable combination of the most suitable optical properties in the structural elements of the light capture module with the functions of the other three, i.e., the water–nutrient flow, gas exchange, and defense–protection module. Under this prism, single structural elements are typically optimized to participate in multiple functions served by different modules (Figure 1). Thus, multitasking structures involved in more than one function were favoured during evolution, because they offer significant economy by means of biomass allocation, and they improve plant fitness [121]. The spatial distribution of the structural elements is usually compatible with their additional functions.

Superficial structural elements are manifold interfaces between the plant and the biotic and abiotic environment. The epidermis is a key structural element, involved not only in the function of the light capture module, but also in the function of the other three modules. This tissue, together with the highly hydrophobic lipid-rich cuticle, protects the leaf against uncontrolled water losses and regulates gas exchange through the stomata (water–nutrient flow and gas exchange modules) [46,122]. It also prevents pathogen and herbivore attacks and provides the mechanical support necessary for the integrity of plant organs (defense–protection module). Moreover, the cuticle prevents the leaching of ions from the mesophyll cells to the environment as well as the uptake of several substances from the outside. Epicuticular waxes are responsible for the maintenance of the water-repellent and self-cleaning properties. Finally, the epidermis integrates complex signals both from the internal tissues and from the external environment, and is also crucial for the development of the expanding lamina [123]. In the case of the lens-like epidermal cells, their suitable shape offers an additional function. Leaves with the above type of epidermal cells typically have an extremely hydrophobic surface, with increased water repellency [33] and reduced presence of fungal and bacterial pathogens [50,124,125]. Epidermal appendages such as non-glandular trichomes also play multiple roles and protect the leaves against biotic (herbivores and pathogens) and abiotic (water losses) stress factors [62].

An important link for these multiple functions of the epidermis and the non-glandular trichomes is the occurrence of the phenolic compounds, i.e., multifunctional compounds that behave not only as UV filters and antioxidants (protection module), but also act as pro-oxidant, antifeeding agents, or toxic factors (defense module) [30,31]). As antioxidants, phenolic compounds located in the epidermis and photosynthetic cells reduce oxidative damage caused by both biotic (pathogens and herbivores) and abiotic stresses (high UV and PAR intensities) [30,126]. Cuticular phenolic compounds also provide mechanical and chemical strength by increased ether- and ester-bond cross-linking [24].

Sclereid idioblasts and lignified BSEs often occur in xeromorphic leaves, possibly playing a role in preventing tissue damage under drought or mechanical stress ([127], see also [128]). Another significant role of both structural elements seems to be the hydraulic integration of the lamina connecting the vascular bundles to the epidermis, thus reducing the resistance in the water path between the veins and stomata [129,130,131,132]. In the case of heterobaric leaves, the formation of areoles in the lamina by BSEs reduces the spread of pathogens and restricts the lateral gas flow, thus allowing for independent gas exchange rates by patchy stomatal opening/closure [133,134]. Hence, BSEs act as a hub integrating leaf mechanics, photosynthetic performance, and hydraulic function [134,135].

Another example of the involvement of a structural element in multiple functions, also served by the light capture module, is the epidermal glands that excrete excess salt in mangroves and halophytes. The secretion of salt through these glands constitutes a significant detoxification mechanism developed mainly to avoid osmotic imbalance and the loss of ionic homeostasis in the photosynthetic tissues [136,137].

Multiple functions have also been proposed for fusoid cells. Except for the trapping and redistribution of light, they are involved in water storage and transportation, contributing to the regulation of the leaf–water balance [138,139].

Lastly, except for their role in light scattering, biominerals such as CaOx crystals and cystoliths represent multifunctional tools that are essential, especially under stress conditions [98]. They share some similar functional characteristics, such as the regulation of Ca level, and the release of CO_2_ and water molecules upon decomposition. The released CO_2_ is assimilated by a low rate photosynthesis called “alarm photosynthesis”, which is essential under drought conditions when the stomata are closed [98,140]. Moreover, phytoliths offer structural support and protection against herbivores [141].

## 5. Acclimatization of Optical Structural Elements to Different Light Regimes

Plant responses to a particular light regime can be considered at different time-scales; seconds (sunflecks), hours (diurnal changes), months (seasonal changes), and years (gap formation in a canopy or forest) [46]. Plants have evolved specific adaptation and acclimatization mechanisms in order to counteract and survive these short- and long-term light fluctuations. Adaptation refers to the inherent structural (as previously mentioned) and functional elements present regardless of the prevailing light regime. Acclimatization refers to the induced structural and functional modulations, including altered gene expression due to environmental cues, especially during growth [142]. The early acclimatization responses to short-term changes in light regime (seconds, hours) are mostly reflected in functional modulations and are usually reversible. Such modulations are, for example, the rapid adjustments of PSII reaction centres related to the distribution and quenching of energy captured in the chloroplasts. Acclimatization responses to long-term changes in the light regime (months, years) is a systemic mechanism that includes irreversible structural, biochemical, and physiological modifications on a whole-plant level and leads to a homeostatic compensation to the specific light regime. An important component of long-term light acclimatization is the expansion of leaves that are more efficient under the particular environment. Obviously, such irreversible structural and, to a lesser extent, biochemical modifications can only occur during leaf development. As a result, mature leaves are unable to undergo such long-term permanent adjustments [46]. The most prominent example of long-term acclimatization responses is the sun (expanded under high light intensities) and shade (expanded under low light intensities) leaves that can occur in the same plant species or, even, the same individual according to different canopy positions. The different light regimes during leaf expansion cause dramatic changes in the anatomical, biochemical, and functional characteristics of all of the structural elements of the leaf, affecting the optical and gas exchange properties and, eventually, photosynthetic capacity [143]. It is also important to note that each modulation, due to the growth environment, affects all functions in which the particular leaf attribute is implicated, considering that most traits are multifunctional [121]. For instance, sun leaves have a higher epidermal flavonoid content (higher UV filtering capacity) and higher adaxial and abaxial epidermal cell wall and cuticle thickness compared with the shade ones [144,145,146]. Moreover, supplemental UV-B radiation causes a significant increase in leaf cuticle thickness and mass (on a unit of leaf area basis) in some Mediterranean plants [147]. These acclimatization changes are targeted at increasing the UV-B filtering capacity of the epidermis and counteracting the UV-B radiation damage, but they also reduce cuticular transpiration [148] and increase the defense potential.

The presence of light conducting elements (the layers of the palisade parenchyma and the density of BSEs or sclereids) is more intense in sun compared with shade leaves. This significant difference is compatible with the light transferring function of these structural elements, as sun leaves are also thicker and light-deficient deep cell layers may occur despite having access to full sunlight compared with shade leaves [119,129]). Probably for the same reason, (a) bifacial leaves change the ratio of palisade to spongy parenchyma cells according to the light regime, with sun leaves having a higher ratio than shade leaves [149]. The higher proportion of palisade cells in the sun leaves is related to the deeper penetration of light, whereas the higher proportion of spongy mesophyll cells in shade leaves increases light scattering and therefore absorptance [5,88,92], and (b) heterobaric sun leaves are characterized by decreased BSE spacing (or higher density) compared with shade leaves [108,150,151]. Thus, light conducting elements take an active part in the plasticity of the leaf structural and functional traits in response to the irradiance levels [152].

The indumentum of mature leaves of many plants is considered as a fixed and static structural element because usually the cells of the trichomes are dead at maturity, hence there is no chance for further structural or biochemical changes [62]. However, as a long-term acclimatization response, trichome layers can change their structural and biochemical characteristics according to the prevailing light regime during development [62,153]. The exposure of developing leaves to high PAR or UV radiation intensities induces an increase in the trichome density ([65], see also [154]), as well as qualitative and quantitative changes in the phenolic content of the individual trichomes [65,68]. Similar results were obtained for the glandular trichomes of *Phillyrea latifolia* [155]. Moreover, under continuous UV-B irradiation, the number of cells and the polyphenolic content of the trichomes increased [156,157].

## 6. Conclusions and Outlook

The evidence from the great variability, both in terms of adaptation (inter-specifically) and acclimatization (intra-specifically or even intra-genotypically), suggests that light capture has been optimized to increase leaf photosynthetic efficiency, but also to protect leaves from high intensities of PAR and UV radiation. The optical properties of virtually all structural elements of leaves are integrated, with many able to undergo considerable modulation so as to be efficient not only in terms of the light capture module, but also functionally incorporated with other functional modules of the leaf entity. This tremendously sophisticated design proves the critical importance of the structural and functional optimization of the leaf optical properties in plant survival and productivity under fluctuating environments.

The optical role of several structural elements (components of the light capture module) is still under debate. For example, the role of non-photosynthesizing tissues in complex optical phenomena (detour effect and sieve effect) is not definite, especially in regards to the geometry of light (collimated or diffuse). The answer to these questions is an important challenge, both because these phenomena are universal, and because it will be useful for plant improvement programs and for planning elements of modern crop systems such as artificial lighting in plant factories, etc. Secondly, understanding the reasons for the notable variety in leaf designs (in terms of adaptation) and plasticity (in terms of acclimatization) on photosynthesis requires long-term research and new analytical, simulating, and modeling tools that will allow us to reconstruct this complexity and elucidate the role of each component and as a whole. A third challenge will come from the necessity to scale-up this cell-, tissue-, and leaf-level structure to function model at larger levels of organization (plant-, canopy-, crop-, and ecosystem-level), as well as to further integrate functional anatomy with photosynthesis and other functions of the leaf (the other three modules). This integration will allow us to predict how plants with specific characteristics behave under particular environments.
